# Melatonin and Prostate Cancer: Anti-tumor Roles and Therapeutic Application

**DOI:** 10.14336/AD.2022.1010

**Published:** 2023-06-01

**Authors:** Mark F Megerian, Jae Seok Kim, Jad Badreddine, Sung Hwi Hong, Lee E Ponsky, Jae Il Shin, Ramy Abou Ghayda

**Affiliations:** ^1^Case Western Reserve University School of Medicine, Cleveland, Ohio, USA.; ^2^ Department of Internal Medicine, Yonsei University Wonju College of Medicine, Wonju, South Korea.; ^3^Urology Institute, University Hospitals Cleveland Medical Center, Case Western Reserve University School of Medicine, Cleveland, Ohio, USA; ^4^Severance Hospital, Yonsei University College of Medicine, Seoul, South Korea.; ^5^Department of Pediatrics, Yonsei University College of Medicine, Seoul, South Korea.

**Keywords:** melatonin, prostate cancer, aging, mechanisms, anti-tumor, oncostasis

## Abstract

Melatonin is an endogenous indoleamine that has been shown to inhibit tumor growth in laboratory models of prostate cancer. Prostate cancer risk has additionally been associated with exogenous factors that interfere with normal pineal secretory activity, including aging, poor sleep, and artificial light at night. Therefore, we aim to expand on the important epidemiological evidence, and to review how melatonin can impede prostate cancer. More specifically, we describe the currently known mechanisms of melatonin-mediated oncostasis in prostate cancer, including those that relate to the indolamine’s ability to modulate metabolic activity, cell cycle progression and proliferation, androgen signaling, angiogenesis, metastasis, immunity and oxidative cell status, apoptosis, genomic stability, neuroendocrine differentiation, and the circadian rhythm. The outlined evidence underscores the need for clinical trials to determine the efficacy of supplemental, adjunct, and adjuvant melatonin therapy for the prevention and treatment of prostate cancer.

## 1. Introduction

Across the world, prostate cancer is the second most frequently diagnosed malignancy and a leading cause of death in men [1]. But, despite many researchers and clinicians' efforts to improve the survival rate of prostate cancer, more advanced treatment strategies are still warranted [2]. The primary treatment for prostate cancer is radical prostatectomy or radiation therapy. Androgen deprivation therapy (ADT) and docetaxel chemotherapy are used for metastatic tumors. Furthermore, targeted therapy has been recently introduced even in prostate cancer treatment [3].

Melatonin (N-Acetyl-5-Methoxytryptamine) is an endogenous indolamine that regulates the sleep-wake cycle. Interestingly, many studies have demonstrated the anti-tumor activities of melatonin. In particular, many in vitro and in vivo studies have described various roles of melatonin in preventing and treating prostate cancer [4]. According to previous research, there are many mechanisms underlying the anti-tumor effects of melatonin. We believe that understanding the anti-tumor mechanism of melatonin could provide insights into the association of circadian rhythm with tumor cell proliferation and apoptosis [5].

This review aims to delineate epidemiological evidence for the association between melatonin and prostate cancer and to review the mechanisms of numerous melatonin-induced oncostatic effects. The outlined evidence underscores the need for clinical trials to determine the efficacy of supplemental, adjunct, and adjuvant melatonin therapy for the prevention and treatment of prostate cancer.

## 2. Melatonin and prostate cancer: epidemiological evidence

Aging is a tenet of mortality that is associated with physiological changes that have internal and external manifestations. Endogenous melatonin is not immune to senescence. In terms of development, melatonin secretion becomes circadian between the age of one to three years and gradually diminishes by 10-15% per decade [6]. Diminished lifetime secretion of melatonin has been suggested to be a catalyst for not only aging [7] (dubbed the “Age Clock” [8]), but also numerous age-related conditions such as cancer [9], neurodegenerative disease (including Alzheimer’s [10] and Parkinson’s disease [11]), type 2 diabetes mellitus, cardiovascular disease, sleep and mood disorders, migraines, and general pain [12, 13]. These diverse ramifications reflect the scores of systemic functionalities of the pleiotropic molecule that might be lost with age and potentially restored with adjunct melatonin supplementation [14, 15].

**Table 1:** Results of epidemiological studies on MT and PC risk.

Author	Year	Study Design	Important Findings/Conclusions
Bartsch et al.	1985	Prospective Cohort	MT circadian rhythm present in young men and patients with benign prostatic hyperplasia and incidental PC, but not present in non-metastasizing PC; Incidental PC patients had elevated 24-hour mean concentration and amplitude compared to PC patients
Feychting et al.	1998	Retrospective Cohort	Lower incidence of all cancers combined in totally blind people (SIR = 0.69; 95% CI = 0.59-0.82); equal risk reduction in hormone-dependent tumors and other types of cancer; lower incidence in severely visually impaired (SIR = 0.95; 95% CI: 0.91-1.00)
Pukkala et al.	2003	Prospective Cohort	Among male commercial airline pilots, increasing number of flight hours increased relative risk of PC in long-distance aircraft (p= 0.01)
Kubo et al.	2006	Prospective Cohort	Rotating-shift workers at significant relative risk for PC compared to dayworkers (RR = 3.0, 95% CI: 1.2-7.7); small and non-significant increase in risk for fixed-night work
Kakizaki et al.	2008	Prospective Cohort	Lower risk of PC associated with increasing sleep duration; men who slept > 9 hours/day at less risk for PC compared to those who slept less (Multivariate HR = 0.48, 95% CI: 0.29-0.79; p = 0.02)
Kloog et al.	2009	Retrospective Cohort	LAN significantly associated with PC, but not colon or lung cancer; PC risk in highest LAN-exposed countries 110% higher compared to lowest LAN-exposed countries
Parent et al.	2012	Case-Control	OR for PC amongst men who ever worked at night compared to those who never worked at night were 2.77 (95% CI: 1.96-3.92)
Sigurdardottir et al.	2015	Case-Cohort	Men who reported sleep problems had lower morning urinary 6-sulfatoxymelatonin (aMT6s) levels; compared to men with morning urinary aMT6s levels above the median, 4-fold increase in risk for advanced PC in men with aMT6s levels below the median (HR: 4.04; 95% CI: 1.26-12.98)
Tai et al.	2016	Case-Control	Less likelihood for PC (aOR = 0.59, 95% CI: 0.35-0.99; aOR = 0.46, 95% CI: 0.27-0.77) or advanced PC (aOR = 0.49, 95% CI: 0.26-0.89; aOR = 0.33, 95% CI = 0.17-0.62) in men with urinary melatonin-sulfate level or melatonin-sulfate/cortisol ratio above the median; men with both low MT/C ratios and PSA level >10 ng/ml at 8.82-fold greater likelihood of PC and 32.06-fold greater likelihood of advanced PC; low MT/C ratio and PSA level > 10ng/ml showed greatest potential in detecting both PC and advanced PC
Kim et al.	2017	Retrospective Cohort	PC incidence associated with artificial LAN (RR = 1.02; p = 0.0369) and urbanization (RR = 1.06, p = 0.0055); comparing PC incidence at 25% and 75% levels of artificial LAN, RR was 1.726 (12.6 over 7.3, respectively); no association between artificial LAN and any other cancer
Farahani et al.	2020	Case-Control	Compared to patients with BPH, patient with PC had significantly lower serum and saliva concentrations of MT (p < 0.05)

BPH: benign prostate hyperplasia; CI: confidence interval; HR: hazard ratio; LAN: light at night; MT: melatonin; MT/C: melatonin/cortisol; OR: odds ratio; aOR: adjusted OR; PC: prostate cancer; PSA: prostate specific antigen; RR: relative risk; SIR: standardized incidence ratio

Prostate cancer is a leading cause of death in men with a 60% incidence in men over the age of 65 [1]. While confirmed diagnoses are significant, a systematic review reporting on autopsies of 6024 men found undiagnosed prostate cancer in 36% and 51% of Caucasians and African Americans aged 70-79, respectively [16], accentuating the true age-related risk of prostate cancer. In addition to this clear age-related risk is an association between prostate cancer risk and diminished levels of endogenous melatonin [17, 18], described further in the next section. Thus, while prostate cancer has a multifactorial etiology, the relationship between advanced age and prostate cancer risk may be related to the steady decline of melatonin that accompanies the aging process. Previous epidemiological studies have identified diminished or suppressed melatonin as an important risk factor for prostate cancer in men. Numerous important studies are summarized in Table 1.

Disruption of the circadian rhythm has been suggested to increase the risk of cancer [19-21]. The International Agency for Research on Cancer of the WHO has associated disruption of circadian rhythm resulting from work shift with possible carcinogenicity in 2007. Several reports have correlated non-standard shift work and shift work sleep disorder with poor health consequences notably on men’s urologic health such as hypogonadal symptoms, infertility, lower urinary tract symptoms, and prostate cancer [22]. An ecological study that involved 164 countries examined the relationship between exposure to light at night and the risk of several types of cancer including prostate cancer [23, 24]. The results showed a significant positive relationship between light exposure at night and the risk of prostate cancer whereby exposure to 99.21 nanowatts/cm2/sr of light at night increased the risk of prostate cancer by 80%. Furthermore, in an attempt to study the relationship between sleep duration and prostate cancer, an epidemiological study was conducted on a Japanese population and a non-significant increase in the risk of prostate cancer was noted in people who sleep less than 6 hours [25].

Kubo et al. examined the effect of shift work on prostate cancer in a prospective cohort study and reported a significant increase in the risk of prostate cancer in rotating shift workers who alternate between a day and/or afternoon shift and a night shift, and smaller non-significant increased risk in fixed-night work when compared to day workers [26]. In a retrospective cohort study conducted in the Nordic countries, pilots aged over 60 years with more than 10,000 block hours had a fourfold increased risk of prostate cancer when compared to pilots with less than 5000 block hours [27]. In addition, the role of circadian genes was investigated in prostate cancer by Zhu et al. in an American population-based case-control study among Caucasian men whereby 17 single nucleotide polymorphisms (SNP)s located in three core genes (i.e., ARNTL, CSNKIE, and NPAS2) were identified and significantly related to prostate cancer [28]. It was also reported that blind people had lower incidences of cancer with a standardized incidence ratio of 0.69 [95% confidence interval (CI) = 0.59-0.82] [29].

The correlation between the urinary metabolites of melatonin and prostate cancer risk was investigated in several case-control studies and found to be an inverse relationship whereby lower levels of urinary metabolites were associated with a higher risk of prostate cancer [30]. Salivary and serum concentrations of melatonin were found to be significantly decreased in patients with prostate cancer when compared to patients with benign prostatic hyperplasia [31]. In addition, Bartsch et al. studied the fluctuation in melatonin and other pituitary hormone levels in elderly men with benign prostatic hyperplasia (BPH), incidental prostatic tumor, and prostate carcinoma [32]. It was found that melatonin exhibited a prominent fluctuation in patients with BPH and incidental carcinoma, a feature not seen in those with prostate carcinomas. Moreover, the levels of melatonin in patients with prostate cancer were reported to be the lowest among the three groups. These results suggest that the levels of melatonin and its normal fluctuation are dysregulated in patients with prostate cancer.

## 3. Oncostatic mechanisms of melatonin

**Figure 1.**
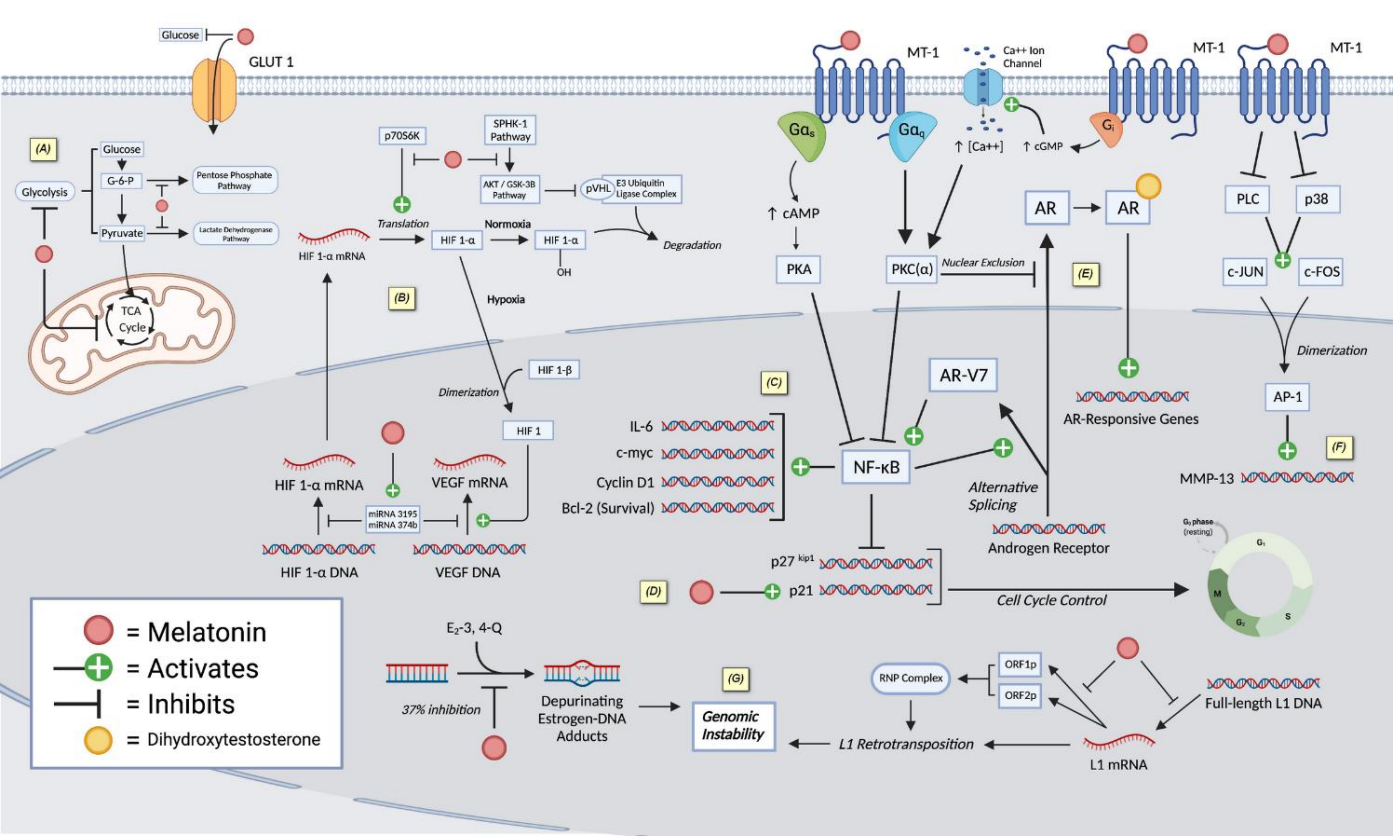
Various described mechanisms of melatonin-induced oncostasis in prostate cancer (created with BioRender.com). Melatonin inhibits (A) glucose metabolism of cancer cells as a competitive inhibitor of GLUT1 and (B) angiogenic processes through inhibiting HIF-1α. Melatonin downregulates NF-kB activities leading to (C) promote apoptosis and (D) inhibit cell proliferation. (E) Melatonin attenuates androgen receptor acitivity through nuclear exclusion of androgen receptor. (F) Melatonin inhibits metastasis by downregulating MMP-13. (G) Melatonin prevents carcinogenesis by reducing genomic instability. AMP: adenosine monophosphate; AP-1: activator protein 1; AR: androgen receptor; ATP: adenosine triphosphate; AR-V7: androgen receptor variant 7; Bcl-2: B-cell lymphoma 2 apoptosis regulator; cGMP: cyclic guanosine monophosphate; c-myc: cellular-myc; E2-3,4-Q: estradiol-3,4-quinone; GLUT1: glucose transporter-1; G-6-P: glucose-6-phosphate; HIF1: hypoxia-inducible factor 1; IL-6: interleukin-6; L1: long interspersed element-1; MMP-13: matrix metallopeptidase 13; MT-1: melatonin receptor 1; NF-kB: nuclear factor kappa-light-chain-enhancer of activated B-cells; PKA: protein kinase A; PKC: protein kinase C; PLC: phospholipase C; pVHL: Von-Hippel Lindau protein; p38: p38 mitogen-activated protein kinases ; p70S6K: ribosomal protein S6 kinase; RNP: ribonucleoprotein complex; TCA Cycle: tricarboxylic acid cycle; VEGF: vascular endothelial growth factor.

Here we describe the currently known mechanisms of melatonin-mediated oncostasis in prostate cancer, including those that relate to the indolamine’s ability to modulate metabolic activity, cell cycle progression and proliferation, androgen signaling, angiogenesis, metastasis, immunity and oxidative cell status, apoptosis, genomic stability, neuroendocrine differentiation, and the circadian rhythm. Many mechanisms are as well depicted in Figure 1.

### 3.1. Oncostasis through the modulation of glucose metabolism

One of the many ways by which tumor cells alter their metabolic activity to sustain survival is to increase the uptake and utilization of glucose. The Warburg Effect describes the reliance of tumor cells on anaerobic respiration through the glycolytic pathway rather than mitochondrial aerobic respiration, even in the presence of oxygen [33]. For this reason, Otto Warburg dubbed this seemingly paradoxical phenomenon aerobic glycolysis in the 1920s [34], and since then, extensive research has elucidated numerous mechanisms of increased glucose uptake and utilization that support this unique metabolic phenotype in tumor cells [35]. In prostate cancer, glucose metabolism is involved in the progression of carcinogenesis [36], where oxidative phosphorylation is active early in disease progression [37], and the Warburg effect takes over in later stages of the disease [38].

The role of melatonin in glucose bioenergetics of prostate cancer has been studied. After previously demonstrating that the major mechanism of melatonin uptake in LNCaP and PC-3 prostate cancer cells were mediated via an active process rather than passive diffusion [39], Hevia et al. later demonstrated a melatonin receptor-independent mechanism of melatonin uptake through members of the glucose transporter (GLUT) family transporters. Specifically, indolamine was found to interact at the same location as GLUT1 and prevented glucose uptake after 30 min, 1, 3, and 6 hours. Intracellular melatonin concentration was diminished as well with the administration of glucose and other known GLUT1 competitive ligands, suggesting competition between melatonin and glucose by the glucose transporter. This competition was then demonstrated in vivo where pharmacological doses of melatonin attenuated the glucose-induced tumor progression and prolonged transgenic adenocarcinoma of the mouse prostate (TRAMP) mice survival [40].

The same research team further examined the specific roles of melatonin in prostate cancer metabolism. Utilizing 13C-labeled metabolites and measuring adenosine triphosphate (ATP)/adenosine monophosphate (AMP) levels and lactate dehydrogenase and pentose phosphate pathway activity, Hevia et al. discovered numerous melatonin-induced metabolic alterations. Not only did they confirm their previous findings that melatonin significantly reduced glucose uptake into prostate cancer cells, but they also found that levels of 13C-labeled lactate, citrate, glutamate, succinate, fumarate, and malate were all decreased secondary to melatonin administration in LNCaP cells, in both normal and hyperglycemic states. In addition, melatonin reduced lactate 13C-labeling, ATP production, and pentose phosphate pathway activity in the androgen-sensitive cells, and reduced lactate 13C-labeling in the androgen-insensitive PC-3 cells. The researchers attributed this salient reduction of all major pathways of glucose metabolism mechanistically to the general reduction of glucose uptake in prostate cancer cells secondary to melatonin administration [37].

This apparent reduction of glucose uptake was further confirmed in vivo by Dauchy et al. Upon showing that daytime spectral transmittance of white light utilizing blue-tinted rodent cages induced a 7-fold increase in circulating melatonin levels [41], Dauchy et al. investigated whether this blue-light amplification of nocturnal melatonin further exhibited oncostatic qualities on prostate cancer xenografts. In male nude rats bearing PC3 prostate cancer xenografts enclosed in blue-tinted rodent cages, numerous metabolic and signaling activities were suppressed secondary to the induced supra-physiologic melatonin levels that slowed tumor growth. Of relevancy, glucose uptake and lactate production were markedly reduced, substantiating melatonin’s suppression of the Warburg effect in prostate cancer [42].

### 3.2. Oncostasis through cell cycle control and anti-proliferation

Many mechanisms have been proposed to explain the antiproliferative effect of melatonin in prostate cancer cells as they relate to the egulation of the cell cycle. Using the radioactive nucleoside 3H-thymidine as an estimate of DNA synthesis and cellular proliferation, Lupowitz et al. demonstrated that melatonin inhibited 3H-thymine incorporation in LNCaP cells at 1-24 hours while transiently attenuating prostate cancer cell growth. The authors suggested melatonin-mediated activation of protein kinase C (PKC) as a possible mechanism of DNA suppression though was not equivocal [43]. Xi et al. not only reproduced melatonin-induced inhibition of 3H-thymidine incorporation in LNCaP cells but also achieved the same end with 2-iodomelatonin, a known agonist of the melatonin receptor, highlighting the involvement of melatonin receptor signaling in this anti-proliferative mechanism [44]. This same group translated their work in vivo with nude mice xenografted with LNCaP cells expressing the melatonin receptor type 1 (MT1), and PC-3 cells void of the receptor. They found LNCaP tumor growth to be significantly attenuated and even prevented, with 51.7% and 38.7% average decreases in tumor volume at 60 days following tumor cell inoculation for mice that received daily melatonin treatment initiated 10-days prior to and following LNCaP inoculation, respectively. The mechanism of oncostasis was deemed to be anti-proliferative, as there were significant decreases in proliferating cell nuclear antigen (PCNA) and cyclin A, the former an integral protein directly involved in DNA replication and repair during the cell cycle [45], and the latter a ubiquitous cyclin that boasts anterograde cell-cycle control via activation of two cyclin-dependent kinases (CDK), CDK1 and CDK2 [46, 47]. Importantly, these same results were not found in the PC-3 xenografted mice void of MT1, pointing to a permissive role of the melatonin receptor in the observed melatonin-mediated anti-proliferative effects [44]. Alternatively, Moretti et al. demonstrated in vitro that via a membrane receptor-independent mechanism, melatonin accumulated LNCaP cells in Go/G1 phase and diminished S-phase, effectively promoting cell cycle withdrawal [48]. This same group later reproduced these exact findings in DU 145 prostate cancer cells, attributing the melatonin-mediated anti-proliferative effects on putative nuclear receptors [49].

Other melatonin receptor-dependent mechanisms of cell cycle control and anti-proliferation have been described. Siu et al. discovered modulation of the epidermal growth factor (EGF) signaling cascade via melatonin activation of MT1. Epidermal growth factor receptor (EGFR) is a notable receptor tyrosine kinase and proto-oncogene complicit in the pathogenesis of numerous carcinomas [50], with EGFR expression found to increase throughout prostate carcinogenesis [51]. Previous research has shown that the EGF/EGFR axis upregulates cyclin D1 in LNCaP cells to promote G1/S progression as a mechanism of proliferation [52]. Siu et al. demonstrated that melatonin inhibited LNCaP proliferation and significantly attenuated cyclin D1 levels, suggesting melatonin - MT1 signaling modulated the EGFR axis as means for antiproliferation [53].

In LNCaP cells, Sainz et al. demonstrated that melatonin induced cell cycle arrest via marked upregulation of p21, a powerful CDK inhibitor [54]. Similarly, Tam et al. demonstrated in 22Rv1 prostate cancer cells that via the MT1, melatonin reduced proliferation through co-activation of parallel PKC and protein kinase A (PKA) cascades that resulted in the upregulation of p27Kip1 [55], another CDK inhibitor with targets such as G1 cyclin E-CDK2 and cyclin D-CDK4/6 [56]. Soon after, they demonstrated this same mechanism in prostate epithelial RWPE-1 cells, suggesting that melatonin can regulate the growth of prostate tissue in both healthy and disease states [57]. Shiu et al. later identified dual activation of Gαs and Gαq as the direct G-protein effectors of MT1 stimulation responsible for initiating signal propagation through parallel PKA and PKC pathways, respectively [58]. Further, after demonstrating that targeted mutation of the nuclear factor kappa-light-chain-enhancer of activated B-cells (NFκB) binding sequence within the 116 base-pair regions of the p27Kip1 promoter in melatonin-treated LNCaP and 22Rv1 cells resulted in diminished binding of NFκB to the p27Kip1 promoter, Shiu et al. suggested NFκB be the specific transcription factor ultimately involved in the melatonin-mediated upregulation of p27Kip1. The likelihood of NFκB involvement was further bolstered after demonstration of both melatonin-mediated inhibitions of NFκB activity via the MT1/PKA (Gαs)/PKC(Gαq) signaling cascade and mimicked and inhibited anti-proliferative activity following treatment of NFκB stimulants and inhibitors, respectively. Thus, they concluded that via the MT1/PKA(Gαs)/PKC(Gαq) signaling pathway, melatonin directly inhibited the constitutively active NFκB in prostate cancer cells, resulting in diminished repression of the p27kip1 promoter, thereby upregulating transcription of p27Kip1 [59]. NFκB inhibition secondary to melatonin treatment in LNCaP and 22Rv1 prostate cancer cells was repeated by Sun Liu et al., further verifying NFκB as a likely downstream target of melatonin [60]. Tmelatonin-mediatedated upregulation of p27Kip1 is salient as most advanced prostate cancers lack p27Kip1 expression [61] and low p27 expression is reported to be an independent prognostic factor for disease recurrence [62]. Interestingly, signaling through the melatonin-mediated PKA(Gαs)/PKC(Gαq)/NFκB/p27Kip1 axis was found by Tam et al. to ultimately be dependent on the presencean of androgen receptor (AR). They proposed the existence of crosstalk between this cascade and AR signaling following observation of an apparent lack of melatonin-mediated anti-proliferation and p27Kip1 upregulation in LNCaP, VCaP, 22Rv1, and RWPE-1 cell lines that had AR and AR variants purged via RNA interference. They thereby inferred the presence of AR as a necessary requisite for signaling through the PKA(Gαs)/PKC (Gαq)/NFκB/p27Kip1 axis that mediates the antiproliferative effect of melatonin on prostate cells [63].

Jung-Hynes et al. studied the antiproliferative effects of melatonin as they relate to interactions with NAD+ dependent sirtuin class III histone deacetylase (SIRT1). In their previous research, Jung-Hynes demonstrated that SIRT1 inhibition precipitated significant antiproliferative effects in vitro on prostate cancer cells, attributed to a permissive increase in FOXO-1 acetylation and transcriptional activation. From these findings, they inferred that SIRT1 is complicit in prostate cancer carcinogenesis [64]. They later found that melatonin demonstrated a level of SIRT1 inhibition comparable to that of nicotinamide and suramin (known inhibitors of SIRT1) and resulted in significant antiproliferation of numerous prostate cancer cell lines. Antiproliferation was then further shown in vivo with TRAMP mice, whereby melatonin administration precipitated a significant decrease in prostate tumorigenesis and levels of Ki-67 and PCNA cellular proliferation markers, attributed to the concomitant SIRT1-inhibition [65]. Further studies are warranted to delineate the putative antiproliferative mechanisms underlying melatonin mediated SIRT1 inhibition.

### 3.3. Oncostasis through the attenuation of androgen receptor activity

Rimler et al. were the first to examine the relationship of melatonin with the AR cascade in prostate cancer. Utilizing LNCaP prostate cancer cells, they found that the melatonin neither competitively inhibited the AR, nor affected steroid binding capacity, but did precipitate nuclear exclusion of the AR that they suggested to explain the attenuated AR activity evident by diminished androgen-induced transcriptional activity and downregulation of AR mRNA [66]. Since this first demonstration, melatonin-mediated nuclear exclusion of the AR in prostate cancer cells has been demonstrated repeatedly [67-69], corroborating the indolamine’s anti-androgen effect on prostate cancer cells. Mechanisms explaining this phenomenon have been identified. Lupowitz et al. found in vitro with PC-3 cells that melatonin induced a salient rise in intracellular cyclic guanosine monophosphate (cGMP), resulting in the activation of PKC and subsequent nuclear exclusion of AR. After melatonin and cGMP-mediated AR nuclear exclusion was inhibited following administration of BAPTA, a membrane-impermeable calcium chelator utilized for its ability to reduce intracellular calcium concentration [70], they concluded that the melatonin-induced rise in cGMP must function to increase intracellular calcium concentration, precipitating the PKC activation that mediates the nuclear exclusion of AR [71]. This same group later examined intracellular translocation patterns of specific PKC isoforms in PC-3-AR cells following melatonin administration to further understand the specific complicit mediators of AR nuclear exclusion. Utilizing membrane-association as a reliable measure of PKC activation [72], they found a seven- to eight-fold increase in membrane association of the PKCα isoform following a 30-minute incubation with melatonin, concluding PKCα to be the specific PKC isoform responsible for the melatonin-mediated AR nuclear exclusion [68].

Interestingly, though Lupowitz et al. described a mechanism whereby melatonin mediated an increase in intracellular calcium concentration in PC-3 cells [71], Xi et al. found that when co-incubated with DHT, pharmacological concentrations of melatonin significantly attenuated the rise in intracellular calcium precipitated by the sex hormone and was associated with a reduction in measurable PSA levels. The authors did not attribute this anti-androgenic result to a MT1/PKC-mediated pathway, and rather hypothesized a melatonin-responsive calmodulin possibly responsible for differential regulation of L- and P/Q-type calcium channels to explain the attenuated intracellular calcium levels [73]. No other studies have studied this proposed, equivocal anti-androgenic mechanism.

Tam et al. further contextualized and verified the role of PKC in melatonin-induced anti-androgenic activity in 22Rv1 prostate cancer cells. Via the MT1, melatonin stimulated Gαq specifically to activate PKC, which precipitated significant inhibition of transactivation of the prostate specific antigen (PSA) promoter [55], a well-known transcriptional target of active AR [74, 75]. Consistently verified and quantified by PSA enhancer-promoter luciferase reporter assays, this PKC-mediated inhibition of AR activity is consistent with the aforementioned mechanisms describing the nuclear exclusion of AR in prostate cancer cells secondary to melatonin treatment.

Surgical or medically-induced ADT is a first-line treatment for prostate cancer [76] due to the poignant role that androgen signaling plays in progression of advanced prostatic cancer [77]. While depletion of endogenous androgen as adjuvant therapy is associated with decreased progression of prostate cancer [78], castration-resistance can often occur, with one study finding disease progression within a median of 18-24 months following initiation of ADT [79, 80]. Castration resistance has a multifactorial etiology [81], but is often defined by oncogenic transformation whereby there is autonomous, androgen-independent activation of the AR axis resulting in the disease progression [82]. One mechanism of such autonomy can be explained by androgen receptor splice variants (AR-Vs), alternatively spliced AR-mRNA variants that lack the androgen-binding domain within the COOH-terminal domain [83]. Since these same AR-Vs retain their NH2-terminal trans-activating domain and DNA-binding domain, AR variants are constitutively active and functionally liberated from androgen dependency [83]. In fact, AR-Vs are significantly expressed in castration resistant prostate cancer (CRPC) compared to hormone-naive prostate cancer [84], with androgen receptor variant 7 (AR-V7) expressed in 75% of CRPC cases following ADT and less than 1% of primary prostate cancer cases [85].

Sun Liu et al. reportedly discovered key interactions of melatonin in the attenuation of AR-V7. They first demonstrated that overexpression of AR-V7 precipitated a 2.7-fold and 1.5-fold increase in expression of NFκB in LNCaP and 22Rv1 prostate cancer cells, respectively, further confirmed by a 2-fold increase in a known downstream NFκB target, interleukin (IL)-6 [60]. They suggested that this activation of the NFκB/IL-6 axis may partly explain the mechanism of AR-V7 induced CRPC as IL-6 is a known pro-inflammatory cytokine complicit in prostate carcinogenesis, specifically involved in numerous processes such as proliferation, apoptosis, angiogenesis, and differentiation [86, 87]. Melatonin treatment in LNCaP prostate cancer cells inhibited IL-6 upregulation and suppressed the NFκB-mediated upregulation of AR-V7 demonstrating how the indolamine can impede AR-V7-mediated carcinogenesis and downregulate the receptor variant entirely. This is direct evidence of melatonin disrupting the positive bidirectional signaling between AR-V7 expression and NFκB activation [60]. These observed effects are consistent with and secondary to the nuanced MT1 signaling cascade that involves co-activation of parallel PKA (via Gαs) and PKC (via Gαq) cascades that ultimately suppress active NFκB in prostate cancer cells, described earlier in the Cell Cycle Control and Anti-Proliferation section.

### 3.4. Oncostasis through anti-angiogenesis

Park et al. studied the anti-angiogenic properties of melatonin as it relates to potential effects on the expression of hypoxia inducible factor-1α (HIF-1 α) and vascular endothelial growth factor (VEGF). The former is the α-subunit of HIF that under both hypoxic (and certain growth-factor stimulated) pathways can translocate to the nucleus to join the constitutively expressed β-subunit to form the functional heterodimeric transcription factor [88], which upregulates several “hypoxia-responsive genes” that promote proliferative ability, angiogenesis, invasion, and other oncogenic process [89]. The latter is a well-described angiogenic growth factor that is a classically upregulated oncogene to support tumor vascularization [90]. Park et al. found that pharmacologic administration of melatonin in DU145 prostate cancer cells reduced HIF-1α levels via reduced expression rather than augmented degradation. This conclusion was substantiated as melatonin administration did not affect either mRNA concentration of HIF-1α, nor the half-life of the active protein. Metabolic labeling assays further indicated that the indolamine downregulated de novo synthesis of HIF-1α. They attributed this mechanism of inhibition to suppression of the 70-kDa ribosomal protein S6 kinase (p70S6K), a mitogen-activated serine/threonine kinase that activates upon phosphorylation of its catalytic, linker, and pseudosubstrate domains [91] and regulates cell cycle progression from Go/G1 to S phase by upregulating translation of mRNA transcripts that possess polypyrimidine tract sequences in their 5’-UTR [92]. Prior research has determined that the 5’UTR- sequence of HIF-1α contains these specific polypyrimidine sequences [93], making it a clear target for p70S6K. Park et al. demonstrated that phosphorylation of p70S6K activation was inhibited secondary to melatonin treatment, which resulted in decreased HIF-1α expression in DU145, PC3, and LNCaP prostate cancer cell lines. Inhibition of phosphorylation-induced p70S6K activation was further confirmed by a negative phosphorylation-status of RPS6 and eIF4B, known downstream targets of p70S6K. Finally, their results showed VEGF expression in prostate cancer cells was reduced secondary to pharmacologic levels of melatonin, which they suggested to be a direct consequence of HIF-1α inhibition [77].

Other mechanisms of melatonin-induced inhibition of HIF-1α in prostate cancer cells have also been studied. Sphingosine kinase 1 (SPHK1) is a key oncogenic enzyme that converts sphingosine to sphingosine 1-phosphate (S1P), a signaling lipid that can promote tumor proliferation, angiogenesis, and inflammation [94]. Prior research has shown that SPHK1/S1P activates a protein kinase B (Akt)/glycogen synthase kinase 3β (GSK-3β) pathway that inactivates the Von Hippel-Lindau (pVHL) protein. The pVHL is a tumor suppressor and substrate-recognition component of the E3 ubiquitin ligase complex that in normoxic conditions, will ubiquitinate hydroxylated HIF-1α leading to its subsequent degradation [95]. Thus, via activation of an Akt/GSK-3β signaling cascade, SPHK1 stabilizes HIF-1α by preventing its degradation through inhibition of pVHL [96, 97]. Cho et al. focused on this SPHK1 pathway in vitro with PC-3 prostate cancer cells, and found that compared to normoxic conditions, SPHK1 expression increased under hypoxic environments with a consistent rise in HIF-1α. They subsequently demonstrated in both PC-3 and LNCaP prostate cancer cell lines that melatonin administration significantly inhibited hypoxia-mediated phosphorylation of Akt and GSK-3β, which they determined was secondary to melatonin-induced inhibition of SPHK-1. They concluded that mechanistically, melatonin inhibits HIF-1α stabilization and nuclear translocation in prostate cancer cells via inactivation of the SPHK-1 and Akt/GSK-3β, culminating in the demonstrated anti-angiogenic activity. They further suggested that this SPHK-1 suppression was related to the known antioxidant potential of melatonin, which can block certain hypoxia-induced ROS signaling cascades [98].

In subsequent research, the same group concluded that an anti-angiogenic mechanism of melatonin in hypoxic PC-3 cells was related to altered expression of certain microRNA (miRNA), which are small molecules of single-stranded, non-coding RNA that play a significant regulatory function by binding to sequences of target mRNA and suppressing protein expression either through mRNA cleavage with subsequent degradation or direct translation repression [99]. In a hypoxic PC-3 cell environment, Sohn et al. found that melatonin significantly upregulated miRNA 3195 and miRNA 374b which corresponded with an attenuation of HIF-1α, HIF-2α, and VEGF mRNA levels. Accordingly, the overexpression of miRNA 3195 and miRNA 374b led to downregulation of VEGF and reduced HIF-1α immuno-fluorescent expression, implying that the miRNA 3195 and miRNA 374b target the HIF-1/2α/VEGF axis. In consideration of this team’s previous work studying the SPHK-1 cascade, they suggested that the observed anti-angiogenic activity of melatonin in hypoxic PC-3 cells may be the sequela of attenuation of the HIF-1α/VEGF/SPHK-1 axis in association with certain miRNAs, including the miRNA 3195 and miRNA 374b [100].

Melatonin’s effect on angiogenesis in prostate cancer was further examined in vivo by Paroni et al., who utilized seven-week-old Foxn1nu/nu male mice xenografted with LNCaP human prostate cancer cells. Interestingly, they found a stark dichotomy in their in vivo experiment compared to the in vitro precedent. They found that expression of HIF-1α and phosphorylation of Akt both increased in melatonin-treated mice versus those treated with saline. In addition, expression of neo-vascularizing agents VEGF and its tyrosine kinase receptor VEGF-R2 were both increased in the melatonin-treated group. These seemingly pro-angiogenic molecular changes occurred in the context of a resultant diminished xenograft micro vessel density, indicating anti-angiogenic activity secondary to the melatonin treatment. The authors acknowledged these mechanistic differences between previous in vitro findings and their in vivo findings, suggesting that the elusive melatonin-induced anti-angiogenic mechanism might induce tumor hypoxia, possibly stimulating activation of Akt and expression of HIF-1α in the LNCaP-xenografted mice. Of note, Paroni et al. as well-found decreased levels of Ki67, a known marker of cellular proliferation [101], which contrasts with previous findings that suggest a direct relationship between Akt activation and Ki67 levels [102]. They concluded that the demonstrated oncostatis of melatonin treated LNCaP-xenografts was not related to its anti-hypoxic role, but rather, secondary to the indolamine’s anti-angiogenic and anti-proliferative roles, corresponding to its antioxidant functions [103]. They noted that melatonin-signaling in prostate cancer must differ in vitro versus in vivo, and these mechanisms warrant further studying.

### 3.5. Oncostasis through anti-metastasis

Metastatic dissemination is common in prostate cancer, with bone being the most frequent site of metastasis (84%), followed by distant lymph nodes (10.6%), liver (10.2%), and thorax (9.1%) [104]. Chen et al. reported that bone metastases occur in 70% of patients with advanced prostate cancer [105], further quantifying the severity of metastasis in prostate cancer. Matrix metalloproteinases (MMP)s, a family of zinc-dependent endopeptidases, are complicit in the metastatic transformation seen in cancer secondary to their involvement with the extracellular matrix [106, 107]. Kleiner et al. delineated three crucial mechanisms by which MMPs augment metastasis of tumor cells; namely, that MMPs can promote invasion through degradation of collagen, laminins, proteoglycans, and other components of the extracellular matrix, that MMPs can promote motility via alteration of the adhesive properties of mobile tumor cells, and that MMPs can activate certain “hidden” carcinogenic properties of two proteins, plasminogen, and laminin-5 [108]. In relation to prostate cancer specifically, expression of MMP-2, -3, -7, -9, and -13 has been identified by Gong et al. in both serum and tumor tissue of patients with prostate cancer, with correlation to progression and metastasis [109]. Wu et al. validated MMPs as therapeutic targets for prostate cancer metastasis by demonstrating inhibition of DU-145 and PC-3 prostate cancer cell migration and invasion upon transfection with miRNA-143, a miRNA that directly targets MMP-13 [110].

Wang et al. demonstrated in vitro that melatonin reduced the invasive potential of DU145 and PC-3 prostate cancer cells ultimately via inhibition of MMP-13. Mechanistically, they identified that signaling via the MT1, melatonin suppressed the phospholipase C (PLC) and p38 mitogen-activated protein kinases (p38) cascades to ultimately block the phosphorylation and activation of c-JUN. Typically, c-JUN dimerizes with c-FOS to form activator protein-1 (AP-1), a transcription factor that is involved in the coordinated expression of MMPs [111]. They thus concluded that melatonin exerts anti-metastatic effects on prostate cancer via MT1 mediated inhibition of the PLC and p38 pathways, resulting in the inactivation of c-Jun with ultimate suppression of MMP-13. Then using SCID mice xenografted with PC-3 cells, they demonstrated that melatonin markedly inhibited MMP-13 expression in prostate tumor specimens and suppressed metastasis to distant organs, further exemplifying in vivo the anti-metastatic effects of melatonin on prostate cancer [112].

### 3.6. Oncostasis through anti-oxidation, anti-inflammation, and modulation of mitochondrial bioenergetics

Many reports have established that oxidative stress and mitochondrial damage are key events in the initiation or progression of tumors, including human prostate cancer. The role of melatonin in modulating mitochondrial activity and function was examined by Tamarindo et al. to investigate its antitumor profile [113]. The indolamine has been shown to decrease the total production of H2O2 by 62% and superoxide anions by 40% in PNT1A cells when compared to controls. In addition, melatonin is suggested to be a possible regulator of mitochondrial activity as evidenced by an enhanced oxidative phosphorylation rate and improved respiratory control ratio in these cells. This observed result was probably related to the inhibitory effect of melatonin on the AKT/mTOR pathway. The study also explored the effect of altered cellular physiology on mitochondrial morphology, whereby it reported that the augmentation of mitochondrial activity led to a decrease in the perimeter and area of the organelle. This outcome is possibly explained by the calculated removal of mitochondria or fractions of the organelle that produce higher than normal levels of Reactive Oxygen Species (ROS) leading to a reduction in mitochondrial dysfunction and oxidative stress. Mitochondrial Bioenergetic Reserve Capacity (MBRC) is commonly used as an indicator of the cell’s ability to respond to stress and to meet heightened energy demands. Melatonin was shown to have no effect on the MRBC when used alone, however its use resulted in a 130% increase in MRBC in PNT1A cells when co-incubated with docosahexaenoic acid [113].

It is widely regarded that ROS mediates multiple cellular processes such as growth, proliferation, and apoptosis. This further highlights the need to regulate their activity and intracellular levels. The dysfunctional regulation of ROS in neoplastic cells is a feature that promotes oxidative stress and oncogenic transformation. The cellular redox environment is modulated by electron donor systems such as the thioredoxin system, a major thiol dependent electron donor system [114]. Thioredoxin (TRX1) is an antioxidant protein that regulates the cellular redox state and communicates with downstream molecules such as apoptosis signal-regulating kinase 1 (ASK1), a member of the mitogen-activated protein kinase (MAPK) family that participates in apoptosis and differentiation of cells. The activity of TRX1 is dependent on its redox state as its oxidized form would activate ASK1 and result in initiating apoptosis. Rodriguez-Garcia et al. investigated the effect of melatonin on the TRX1 system and concluded that it had no effect on the basal redox state in LNCap cells, however, it further reduced TRX in PC-3 cells. In addition, LNCap and PC-3 cells were stained with dihydroethidium (DHE) and MitoSOX red probes to monitor for any changes in ROS levels after incubation with melatonin. The results showed a significant decrease in MitoSOX fluorescence and no effect on DHE fluorescence indicating a reduction in ROS production and oxidative stress levels. Further investigations were conducted to evaluate the effect of melatonin on the levels of antioxidant enzymes, and upregulation of CuZnSOD and catalase was noted in western blots [114]. Another study done by Sainz et al. further highlighted the antioxidant property of melatonin by showing reduced levels of DHE fluorescence in LNCap cells after the addition of AAPH to the culture [54]. This effect was less significant with PC-3 cells. These results clearly demonstrate the antioxidant capacity of melatonin either through directly scavenging free radicals or through the upregulation of antioxidant enzymes such as glutathione, superoxide dismutase, and glutathione peroxidase [115].

Many studies have recently shed light on the role of the immune system in augmenting the body’s response against malignant neoplasms through the cytotoxic activity of immune cells and inhibitory properties of cytokines. A depressed immune response has been proposed to be a common feature among cancer patients, an effect attributed to the release of immune-suppressive molecules and the activation of suppressor macrophages. This further suggests that immunomodulation can be an effective therapeutic strategy to boost the body’s response against tumors. Neri et al. examined the effect of melatonin therapy on the immune system in 31 patients with advanced solid tumors, including prostate [116]. After 3 months of treatment, the performance status and quality of life were reported to be improved in a large percentage of the patients. In addition, the cytokine levels measured one month after the onset of treatment revealed an increase in the levels of TNF-alpha by 22%, IL-1 by 38%, IL-2 by 41%, IFN-gamma by 34%, and a decrease in the levels of IL-6 by 70%. It is believed that the modulation of the cytokine system by melatonin mediates the activation and recruitment of the cytotoxic monocytes, T-cells, and NK cells which ultimately enhance host defense against tumors.

Melatonin has also demonstrated an anti-inflammatory property through its inhibitory effect on IL-17 mediated expression of inflammatory cytokines and chemokines as evaluated by Ge et al. [117]. This outcome was mediated by melatonin’s specific action on the Akt-GSK3b pathway whereby an inhibition of Akt ultimately lead to an increased activity of GSK3b, an intrinsic negative regulator of IL-17 mediated inflammatory response. This conclusion was supported by the significant decrease in the mRNA levels of known downstream targets of IL-17 (CXCL1, CCL20, and IL-6) in normal mice prostatic tissue when treated with melatonin. In addition, lower levels of CXCL1 and CCL20 mRNA were detected in PC-3 xenograft tumor perfused with nighttime blood when compared to daytime blood. Other suggested anti-inflammatory mechanisms demonstrated by melatonin include downregulation of cyclooxygenase enzymes and upregulation of anti-inflammatory cytokines.

### 3.7. Oncostasis through pro-apoptosis

Sainz et al. examined the role of antioxidants, namely melatonin, in enhancing apoptosis of androgen-sensitive and insensitive prostate cancer cells induced by tumor necrosis factor and chemotherapeutic gamma radiation [118]. Results demonstrated that pharmacological levels of melatonin precipitated an overall reduction of the number of prostate cancer cells, true for both androgen sensitive LNCaP and androgen insensitive PC3 cells, with the former being most sensitive. Following a 6-day culture, there was significant upregulation of p21, a potent cyclin-dependent kinase inhibitor. Human recombinant tumor necrosis factor-alpha (hrTNFα) treatment demonstrated a dose-dependent reduction of LNCaP cell viability, and when co-incubated with melatonin, resulted in a significant increase in apoptotic cell death. This was not true for PC3 cells, which demonstrated resistance to hrTNFα therapy, both in isolation and in conjunction with adjuvant melatonin. Mechanistically, they demonstrated melatonin inhibited hrTNFα-induced activation of NFκB [54], a transcription factor that may promote prostate cancer viability via upregulation of c-myc, cyclin D1, and IL-6, and more relevantly, activation of anti-apoptotic genes, such as B-cell lymphoma 2 apoptosis regulator (Bcl-2) [119]. This melatonin-induced inhibition was exemplified following a 48-hour LNCaP co-culture with hrTNF-α and melatonin, which demonstrated that melatonin promoted prostate cancer cell apoptosis through reduction of Bcl-2 as well as Survivin levels, two important carcinogenic survival factors. Melatonin failed to enhance γ-radiation-induced apoptosis in prostate cancer cells, attributed by the researchers to the significant melatonin-mediated rise in reduced glutathione (GSH) [54].

Other mechanisms behind the melatonin-mediated apoptotic death of LNCaP cells have been studied. Previous research has suggested that the c-JUN N-terminal kinase (JNK) and p38 kinase are key MAPKs that can mediate pro-apoptotic processes [120], while extracellular signal-regulated kinase (ERK) is widely associated with anti-apoptotic functions [121]. However, there is evidence that that each MAPK can play both pro- and anti-apoptotic roles depending on cell type, stimulus type and strength, and environment [122]. Soo Joo et al. focused on the modulation of the MAPK superfamily by melatonin in relation to prostatic cancer cell survival and apoptosis [123]. Experimenting in vitro with a LNCaP cell line, the JNK and p38 kinases were significantly activated following 6-to-12-hour incubation with melatonin, and ERK was inactivated following 48-hour incubation with melatonin. As well, melatonin administration precipitated a relative increase in Bax and Cytochrome C, two important pro-apoptotic mediators, and a decrease in the cytosolic Bcl-2 survival proteins. They concluded that the pro-apoptotic role of the indolamine seen in the LNCaP cell line was directly dependent on JNK and p38 activation, and independent of ERK signaling cascades. While these specific pro-apoptotic mechanisms have been described in vitro, Xi et al. found no significant increase in vivo with xenografted LNCaP tumor apoptotic cells, instead suggesting the observed melatonin-induced tumor suppression to be secondary to an anti-proliferative mechanism [44].

### 3.8. Oncostasis through the maintenance of genomic stability

Catechol estrogen-3,4- quinones (CE-3,4-Q) are carcinogenic metabolites of estrogens that have been shown to react with DNA to form depurinating adducts that can increase the likelihood for mutagenesis due to the error prone base-excision DNA damage repair mechanism. This sequela imposed by CE-3,4-Q has been proposed to be an initiating factor in the carcinogenesis of many cancers, including breast and prostate [124]. Interestingly, catechol estrogen quinones have been found to be present in urine samples in prostate cancer patients, and thus can be considered an early biomarker in gauging prostate cancer risk [125]. Zahid et al. experimented with numerous natural agents in their ability to prevent estradiol-3,4-quinone (E2-3,4-Q) induced DNA adduct formation, and found that melatonin exerted its inhibitory effects through the reduction of E2-3,4-semiquinone to 4-OHE2, boasting a 37% inhibition of adduct formation [126].

Long interspersed nuclear element-1 (LINE-1) belongs to a family of retrotransposons that can alter genomic stability through insertions via retrotransposition of a RNA molecule into the genome [127]. LINE-1 expression is tightly repressed by numerous mechanisms, including DNA methylation, histone modification, and RNA interference [128, 129]. The regulation of LINE-1 is to promote genomic stability, and thus, dysregulation of LINE-1 has been linked to the carcinogenesis process [130, 131], with one study demonstrating that nearly half of human cancers are immunoreactive for ORF1p, a protein encoded by LINE-1 [132]. ORF1p and ORF2p, the two protein products of LINE-1, are functionally integral for LINE-1 genome integration. deHaro et al. demonstrated both in vivo with PC-3 xenografted adult male nude rats and in vitro with PC-3 cell culture that melatonin diminishes LINE-1 mRNA and ORF1p levels and inhibits LINE-1 retrotransposition in prostate cancer cells [133]. Observation that melatonin receptor antagonist administration increased LINE-1 mobilization in a dose-dependent manner confirmed that this effect was mediated through the MT1. Thus, circulating melatonin’s enhances genomic integrity by inhibiting the deleterious and potentially carcinogenic effect of LINE-1 in prostatic cells.

### 3.9. Oncostasis via neuroendocrine differentiation

Neuroendocrine (NE) cells comprise ~1% of the epithelial compartment of the prostate gland, significantly overshadowed by the predominant luminal and basal cells [134]. While prevalence of NE cells in benign prostate tissue is minimal, they contribute to epithelial cell growth and differentiation [135] via the secretion of certain mitogenic and survival factors in a paracrine manner, such as bombesin, neurotensin, PTHrP, serotonin, and calcitonin [136]. Notably, NE cells lack AR and are thus void of androgen dependence [137], a precarious feature that becomes accentuated in the carcinogenesis of prostate cancer and ultimately the development of CRPC. In fact, neuroendocrine prostatic carcinoma (NEPC) is both highly resistant to and a possible consequence of androgen-deprivation therapy secondary to its androgen-independent phenotype [138, 139]. NEPC represents the deadliest and most aggressive subset of prostate cancer (5-year overall survival of 12.6%) [140], with 17-30% of metastatic CRPC exhibiting small cell neuroendocrine carcinoma (SCNC) histology [141].

While the negative implications of NE differentiation in prostate cancer clinically are unequivocal, melatonin demonstrates a peculiar role in prostate cancer NE differentiation in vitro. Utilizing pharmacological concentrations of melatonin, Sainz et al. demonstrated significant suppression of LNCaP and PC3 cell growth while concomitantly enhancing neuroendocrine differentiation of both the androgen-sensitive and insensitive prostate cancer cells [142]. While levels of cyclic AMP transiently rose following melatonin administration, the underlying mechanism of differentiation was not attributed to PKA, a conclusion substantiated by the failure of H89, a PKA inhibitor, to inhibit differentiation. NE differentiation may be related to the melatonin-mediated upregulation of manganese superoxide dismutase (MnSOD), a mitochondrial superoxide dismutase demonstrated by Quirós et al. to be involved in NE differentiation in LNCaP cells [143]. Mayo et al. further delineated the relationship between redox signaling and NE differentiation, demonstrating a melatonin-mediated increase in GSH upstream of persistent ERK1/2 activation that precipitated ultimate NE differentiation [69]. Further, following incubation of LNCaP cells with melatonin, genomic microarray found the indolamine to upregulate insulin-like growth factor-binding protein 3 (IGFBP3), a gene target determined to underlie the melatonin-mediated NE differentiation of prostate cancer cells. Consistently, chronic melatonin treatment in TRAMP models precipitated a salient rise in serum IGFB3 and correlated with a 33% increased survival. So, while clinically, NE differentiation in prostate cancer is associated with poor prognosis and increased mortality, melatonin-mediated NE differentiation precipitates a certain degree of oncostasis in the laboratory [144]. In fact, Rodriguez-Garcia demonstrated that NE-like cells induced by chronic melatonin treatment displayed greater sensitivity to TNF-alpha and TRAIL than PC-3 and LNCaP cells, suggesting a permissive effect of NE differentiation that enhances efficacy of cytokine induced apoptosis [145].

### 3.10. Oncostasis through the resynchronization of circadian rhythm

The “master clock” of endogenous circadian rhythms is located in the suprachiasmatic nucleus of the anterior hypothalamus. Intracellularly, circadian rhythms are regulated by the oscillatory transcription and translation of certain genes. Bma11 and Clock, two basic-helix-loop-helix (bHLH)-PAS proteins, dimerize to form a transcription factor complex that regulates the rhythmic transcription of three Period genes (Per1, Per2, Per3), and two Cryptochrome genes (Cry1, Cry2). These products then directly inhibit the Bma11-Clock heterodimer activity via negative-feedback regulation [146]. These core clock components are carefully regulated to maintain the 24-hour rhythmic period. Jung-Hynes et al. demonstrated that compared to normal prostatic epithelial cells, Clock and Per2 protein levels were downregulated while Bma11 was upregulated in multiple prostatic cancer cell lines (LNCaP, 22Rv1, DU145, and PC3) [147]. These exact perturbations were similarly found in human prostate cancer tissue, exemplifying that dysregulated clock circuitry component expression profiles are associated with prostatic cancer. In vitro administration of melatonin resulted in an increase in Clock and Per2 levels and a concurrent decrease in Bma11, effectively “re-synchronizing” the once dysregulated circadian rhythm circuitry seen in the prostate cancer cells. They suggest that the melatonin-induced resynchronization of clock components was related to prostate cancer attenuation, though acknowledge that more studies are warranted.

## 4. Possibilities and limits of melatonin for the treatment of cancer

Although melatonin has various oncostatic effects, it is not yet a standard treatment for malignant tumors, including prostate cancer. Instead, melatonin therapy can alleviate cancer-associated symptoms such as fatigue, anxiety, and insomnia [148, 149]. Many cancer patients suffer from psychological problems such as anxiety and depression, and insomnia. The sleep hormone melatonin can help reduce the stress and insomnia of cancer patients through medication or diet [150]. In addition, several studies demonstrated that melatonin could reduce chemotherapy-associated side effects [151]. In this respect, melatonin can be an effective palliative treatment in cancer patients. However, the role of melatonin as an anti-tumor agent is limited. No clinical trials could demonstrate the significant inhibitory effect of melatonin on tumor progression as a single treatment [152]. Some clinical studies showed the anti-tumor effect of melatonin in combination with chemotherapy [152-155], but not for prostate cancer. A recent randomized controlled trial reported the effect of melatonin on disease-free survival in lung cancer. In the study, melatonin treatment showed no benefit in 2 and 5-year disease-free survival. But, in advanced lung cancer with stage 3 or 4, melatonin showed 5-year disease-free survival benefit [156]. In previous clinical trials that investigated the anti-tumor effects of melatonin, the maintenance dose was mostly 20 mg per day and the duration varied from several weeks to some years [155]. Considering that there were no major side effects related to melatonin except for minor psychological or neurocognitive problems [157], we recommend a higher dosage for a definite anti-tumor activity.

## 5. Conclusion

This review summarizes the numerous cellular mechanisms by which melatonin demonstrates anti-tumor activity in prostate cancer. More specifically, we describe those mechanisms that relate to the indolamine’s ability to modulate prostate cancer cell metabolic activity, cell cycle progression and proliferation, androgen signaling, angiogenesis, metastasis, immunity and oxidative cell status, apoptosis, genomic stability, neuroendocrine differentiation, and the circadian rhythm. Further in vitro and in vivo studies are necessary to not only validate these mechanisms, but also to discover additional mechanisms not yet described in the literature. Finally, while these anti-tumor properties have been demonstrated in laboratory models of prostate cancer, future clinical trials are necessary to determine the efficacy of supplemental, adjunct, adjuvant melatonin therapy for the prevention and treatment of prostate cancer.

## References

[1] Rawla P (2019). Epidemiology of Prostate Cancer. World J Oncol, 10:63-89. 31068988 10.14740/wjon1191 PMC6497009

[2] Silberstein JL, Pal SK, Lewis B, Sartor O (2013). Current clinical challenges in prostate cancer. Transl Androl Urol, 2:122-136. 26816735 10.3978/j.issn.2223-4683.2013.09.03 PMC4708189

[3] Crawford ED, Schellhammer PF, McLeod DG, Moul JW, Higano CS, Shore N, et al. (2018). Androgen Receptor Targeted Treatments of Prostate Cancer: 35 Years of Progress with Antiandrogens. J Urol, 200:956-966. 29730201 10.1016/j.juro.2018.04.083

[4] Anim-Koranteng C, Shah HE, Bhawnani N, Ethirajulu A, Alkasabera A, Onyali CB, et al. (2021). Melatonin-A New Prospect in Prostate and Breast Cancer Management. Cureus, 13:e18124. 34692334 10.7759/cureus.18124 PMC8525668

[5] Cortes-Hernandez LE, Eslami SZ, Dujon AM, Giraudeau M, Ujvari B, Thomas F, et al. (2020). Do malignant cells sleep at night? Genome Biol, 21:276. 33183336 10.1186/s13059-020-02179-w PMC7659113

[6] Grivas TB, Savvidou OD (2007). Melatonin the "light of night" in human biology and adolescent idiopathic scoliosis. Scoliosis, 2:6. 17408483 10.1186/1748-7161-2-6 PMC1855314

[7] Sack RL, Lewy AJ, Erb DL, Vollmer WM, Singer CM (1986). Human melatonin production decreases with age. J Pineal Res, 3:379-388. 3783419 10.1111/j.1600-079x.1986.tb00760.x

[8] Bubenik GA, Konturek SJ (2011). Melatonin and aging: prospects for human treatment. J Physiol Pharmacol, 62:13-19. 21451205

[9] Li Y, Li S, Zhou Y, Meng X, Zhang JJ, Xu DP, et al. (2017). Melatonin for the prevention and treatment of cancer. Oncotarget, 8:39896-39921. 28415828 10.18632/oncotarget.16379 PMC5503661

[10] Hossain MF, Wang N, Chen R, Li S, Roy J, Uddin MG, et al. (2021). Exploring the multifunctional role of melatonin in regulating autophagy and sleep to mitigate Alzheimer's disease neuropathology. Aging Res Rev, 67:101304. 10.1016/j.arr.2021.101304 33610813

[11] Tamtaji OR, Reiter RJ, Alipoor R, Dadgostar E, Kouchaki E, Asemi Z (2020). Melatonin and Parkinson Disease: Current Status and Future Perspectives for Molecular Mechanisms. Cell Mol Neurobiol, 40:15-23. 31388798 10.1007/s10571-019-00720-5 PMC11448849

[12] Hardeland R (2012). Melatonin in aging and disease -multiple consequences of reduced secretion, options and limits of treatment. Aging Dis, 3:194-225. 22724080 PMC3377831

[13] Lahiri DK, Chen D, Lahiri P, Rogers JT, Greig NH, Bondy S (2004). Melatonin, metals, and gene expression: implications in aging and neurodegenerative disorders. Ann N Y Acad Sci, 1035:216-230. 15681810 10.1196/annals.1332.014

[14] Tordjman S, Chokron S, Delorme R, Charrier A, Bellissant E, Jaafari N, et al. (2017). Melatonin: Pharmacology, Functions and Therapeutic Benefits. Curr Neuropharmacol, 15:434-443. 28503116 10.2174/1570159X14666161228122115 PMC5405617

[15] Reiter RJ, Rosales-Corral SA, Tan DX, Acuna-Castroviejo D, Qin L, Yang SF, et al. (2017). Melatonin, a Full Service Anti-Cancer Agent: Inhibition of Initiation, Progression and Metastasis. Int J Mol Sci, 18. 10.3390/ijms18040843 PMC5412427 28420185

[16] Jahn JL, Giovannucci EL, Stampfer MJ (2015). The high prevalence of undiagnosed prostate cancer at autopsy: implications for epidemiology and treatment of prostate cancer in the Prostate-specific Antigen-era. Int J Cancer, 137:2795-2802. 25557753 10.1002/ijc.29408 PMC4485977

[17] Bazzi LA, Sigurdardottir LG, Sigurdsson S, Valdimarsdottir U, Torfadottir J, Aspelund T, et al. (2021). Exploratory assessment of pineal gland volume, composition, and urinary 6-sulfatoxymelatonin levels on prostate cancer risk. Prostate, 81:487-496. 33860950 10.1002/pros.24130 PMC8194005

[18] Sigurdardottir LG, Markt SC, Rider JR, Haneuse S, Fall K, Schernhammer ES, et al. (2015). Urinary melatonin levels, sleep disruption, and risk of prostate cancer in elderly men. Eur Urol, 67:191-194. 25107635 10.1016/j.eururo.2014.07.008 PMC4318783

[19] Parent ME, El-Zein M, Rousseau MC, Pintos J, Siemiatycki J (2012). Night work and the risk of cancer among men. Am J Epidemiol, 176:751-759. 23035019 10.1093/aje/kws318

[20] Kim KY, Lee E, Kim YJ, Kim J (2017). The association between artificial light at night and prostate cancer in Gwangju City and South Jeolla Province of South Korea. Chronobiol Int, 34:203-211. 27996309 10.1080/07420528.2016.1259241

[21] Sigurdardottir LG, Valdimarsdottir UA, Fall K, Rider JR, Lockley SW, Schernhammer E, et al. (2012). Circadian disruption, sleep loss, and prostate cancer risk: a systematic review of epidemiologic studies. Cancer Epidemiol Biomarkers Prev, 21:1002-1011. 22564869 10.1158/1055-9965.EPI-12-0116 PMC3392423

[22] Deng N, Kohn TP, Lipshultz LI, Pastuszak AW (2018). The Relationship Between Shift Work and Men's Health. Sex Med Rev, 6:446-456. 29371140 10.1016/j.sxmr.2017.11.009

[23] Reiter RJ, Tan DX, Korkmaz A, Erren TC, Piekarski C, Tamura H, et al. (2007). Light at night, chronodisruption, melatonin suppression, and cancer risk: a review. Crit Rev Oncog, 13:303-328. 18540832 10.1615/critrevoncog.v13.i4.30

[24] Kloog I, Haim A, Stevens RG, Portnov BA (2009). Global co-distribution of light at night (LAN) and cancers of prostate, colon, and lung in men. Chronobiol Int, 26:108-125. 19142761 10.1080/07420520802694020

[25] Kakizaki M, Inoue K, Kuriyama S, Sone T, Matsuda-Ohmori K, Nakaya N, et al. (2008). Sleep duration and the risk of prostate cancer: the Ohsaki Cohort Study. Br J Cancer, 99:176-178. 18542076 10.1038/sj.bjc.6604425 PMC2453016

[26] Kubo T, Ozasa K, Mikami K, Wakai K, Fujino Y, Watanabe Y, et al. (2006). Prospective cohort study of the risk of prostate cancer among rotating-shift workers: findings from the Japan collaborative cohort study. Am J Epidemiol, 164:549-555. 16829554 10.1093/aje/kwj232

[27] Pukkala E, Aspholm R, Auvinen A, Eliasch H, Gundestrup M, Haldorsen T, et al. (2003). Cancer incidence among 10,211 airline pilots: a Nordic study. Aviat Space Environ Med, 74:699-706. 12862322

[28] Zhu Y, Stevens RG, Hoffman AE, Fitzgerald LM, Kwon EM, Ostrander EA, et al. (2009). Testing the circadian gene hypothesis in prostate cancer: a population-based case-control study. Cancer Res, 69:9315-9322. 19934327 10.1158/0008-5472.CAN-09-0648 PMC2955869

[29] Feychting M, Osterlund B, Ahlbom A (1998). Reduced cancer incidence among the blind. Epidemiology, 9:490-494. 9730026

[30] Tai SY, Huang SP, Bao BY, Wu MT (2016). Urinary melatonin-sulfate/cortisol ratio and the presence of prostate cancer: A case-control study. Sci Rep, 6:29606. 27387675 10.1038/srep29606 PMC4937372

[31] Farahani H, Alaee M, Amri J, Baghinia MR, Rafiee M (2020). Serum and Saliva Concentrations of Biochemical Parameters in Men with Prostate Cancer and Benign Prostate Hyperplasia. Lab Med, 51:243-251. 32374392 10.1093/labmed/lmz053

[32] Bartsch C, Bartsch H, Fluchter SH, Attanasio A, Gupta D (1985). Evidence for modulation of melatonin secretion in men with benign and malignant tumors of the prostate: relationship with the pituitary hormones. J Pineal Res, 2:121-132. 2420960 10.1111/j.1600-079x.1985.tb00633.x

[33] Jones W, Bianchi K (2015). Aerobic glycolysis: beyond proliferation. Front Immunol, 6:227. 26029212 10.3389/fimmu.2015.00227 PMC4432802

[34] Warburg O (1925). The metabolism of carcinoma cells. The Journal of Cancer Research, 9:148-163.

[35] Liberti MV, Locasale JW (2016). The Warburg Effect: How Does it Benefit Cancer Cells? Trends Biochem Sci, 41:211-218. 26778478 10.1016/j.tibs.2015.12.001 PMC4783224

[36] Singh G, Lakkis CL, Laucirica R, Epner DE (1999). Regulation of prostate cancer cell division by glucose. J Cell Physiol, 180:431-438. 10430183 10.1002/(SICI)1097-4652(199909)180:3<431::AID-JCP14>3.0.CO;2-O

[37] Hevia D, Gonzalez-Menendez P, Fernandez-Fernandez M, Cueto S, Rodriguez-Gonzalez P, Garcia-Alonso JI, et al. (2017). Melatonin Decreases Glucose Metabolism in Prostate Cancer Cells: A (13)C Stable Isotope-Resolved Metabolomic Study. Int J Mol Sci, 18. 10.3390/ijms18081620 PMC5578012 28933733

[38] Eidelman E, Twum-Ampofo J, Ansari J, Siddiqui MM (2017). The Metabolic Phenotype of Prostate Cancer. Front Oncol, 7:131. 28674679 10.3389/fonc.2017.00131 PMC5474672

[39] Hevia D, Sainz RM, Blanco D, Quiros I, Tan DX, Rodriguez C, et al. (2008). Melatonin uptake in prostate cancer cells: intracellular transport versus simple passive diffusion. J Pineal Res, 45:247-257. 18341516 10.1111/j.1600-079X.2008.00581.x

[40] Hevia D, Gonzalez-Menendez P, Quiros-Gonzalez I, Miar A, Rodriguez-Garcia A, Tan DX, et al. (2015). Melatonin uptake through glucose transporters: a new target for melatonin inhibition of cancer. J Pineal Res, 58:234-250. 25612238 10.1111/jpi.12210

[41] Dauchy RT, Dauchy EM, Hanifin JP, Gauthreaux SL, Mao L, Belancio VP, et al. (2013). Effects of spectral transmittance through standard laboratory cages on circadian metabolism and physiology in nude rats. J Am Assoc Lab Anim Sci, 52:146-156. 23562097 PMC3624782

[42] Dauchy RT, Hoffman AE, Wren-Dail MA, Hanifin JP, Warfield B, Brainard GC, et al. (2015). Daytime Blue Light Enhances the Nighttime Circadian Melatonin Inhibition of Human Prostate Cancer Growth. Comp Med, 65:473-485. 26678364 PMC4681241

[43] Lupowitz Z, Zisapel N (1999). Hormonal interactions in human prostate tumor LNCaP cells. J Steroid Biochem Mol Biol, 68:83-88. 10215041 10.1016/s0960-0760(98)00164-2

[44] Xi SC, Siu SW, Fong SW, Shiu SY (2001). Inhibition of androgen-sensitive LNCaP prostate cancer growth in vivo by melatonin: association of antiproliferative action of the pineal hormone with mt1 receptor protein expression. Prostate, 46:52-61. 11170132 10.1002/1097-0045(200101)46:1<52::aid-pros1008>3.0.co;2-z

[45] Essers J, Theil AF, Baldeyron C, van Cappellen WA, Houtsmuller AB, Kanaar R, et al. (2005). Nuclear dynamics of PCNA in DNA replication and repair. Mol Cell Biol, 25:9350-9359. 16227586 10.1128/MCB.25.21.9350-9359.2005 PMC1265825

[46] Bendris N, Lemmers B, Blanchard JM, Arsic N (2011). Cyclin A2 mutagenesis analysis: a new insight into CDK activation and cellular localization requirements. PLoS One, 6:e22879. 21829545 10.1371/journal.pone.0022879 PMC3145769

[47] Yam CH, Fung TK, Poon RY (2002). Cyclin A in cell cycle control and cancer. Cell Mol Life Sci, 59:1317-1326. 12363035 10.1007/s00018-002-8510-y PMC11337442

[48] Moretti RM, Marelli MM, Maggi R, Dondi D, Motta M, Limonta P (2000). Antiproliferative action of melatonin on human prostate cancer LNCaP cells. Oncol Rep, 7:347-351. 10671684

[49] Marelli MM, Limonta P, Maggi R, Motta M, Moretti RM (2000). Growth-inhibitory activity of melatonin on human androgen-independent DU 145 prostate cancer cells. Prostate, 45:238-244. 11074526 10.1002/1097-0045(20001101)45:3<238::aid-pros6>3.0.co;2-w

[50] Normanno N, De Luca A, Bianco C, Strizzi L, Mancino M, Maiello MR, et al. (2006). Epidermal growth factor receptor (EGFR) signaling in cancer. Gene, 366:2-16. 16377102 10.1016/j.gene.2005.10.018

[51] Di Lorenzo G, Tortora G, D'Armiento FP, De Rosa G, Staibano S, Autorino R, et al. (2002). Expression of epidermal growth factor receptor correlates with disease relapse and progression to androgen-independence in human prostate cancer. Clin Cancer Res, 8:3438-3444. 12429632

[52] Perry JE, Grossmann ME, Tindall DJ (1998). Epidermal growth factor induces cyclin D1 in a human prostate cancer cell line. Prostate, 35:117-124. 9568675 10.1002/(sici)1097-0045(19980501)35:2<117::aid-pros5>3.0.co;2-g

[53] Siu SW, Lau KW, Tam PC, Shiu SY (2002). Melatonin and prostate cancer cell proliferation: interplay with castration, epidermal growth factor, and androgen sensitivity. Prostate, 52:106-122. 12111702 10.1002/pros.10098

[54] Sainz RM, Reiter RJ, Tan DX, Roldan F, Natarajan M, Quiros I, et al. (2008). Critical role of glutathione in melatonin enhancement of tumor necrosis factor and ionizing radiation-induced apoptosis in prostate cancer cells in vitro. J Pineal Res, 45:258-270. 18384530 10.1111/j.1600-079X.2008.00585.x

[55] Tam CW, Mo CW, Yao KM, Shiu SY (2007). Signaling mechanisms of melatonin in antiproliferation of hormone-refractory 22Rv1 human prostate cancer cells: implications for prostate cancer chemoprevention. J Pineal Res, 42:191-202. 17286752 10.1111/j.1600-079X.2006.00406.x

[56] Lloyd RV, Erickson LA, Jin L, Kulig E, Qian X, Cheville JC, et al. (1999). p27kip1: a multifunctional cyclin-dependent kinase inhibitor with prognostic significance in human cancers. Am J Pathol, 154:313-323. 10027389 10.1016/S0002-9440(10)65277-7 PMC1850003

[57] Tam CW, Chan KW, Liu VW, Pang B, Yao KM, Shiu SY (2008). Melatonin as a negative mitogenic hormonal regulator of human prostate epithelial cell growth: potential mechanisms and clinical significance. J Pineal Res, 45:403-412. 18637986 10.1111/j.1600-079X.2008.00608.x

[58] Shiu SY, Pang B, Tam CW, Yao KM (2010). Signal transduction of receptor-mediated antiproliferative action of melatonin on human prostate epithelial cells involves dual activation of Galpha(s) and Galpha(q) proteins. J Pineal Res, 49:301-311. 20695976 10.1111/j.1600-079X.2010.00795.x

[59] Shiu SY, Leung WY, Tam CW, Liu VW, Yao KM (2013). Melatonin MT1 receptor-induced transcriptional up-regulation of p27(Kip1) in prostate cancer antiproliferation is mediated via inhibition of constitutively active nuclear factor kappa B (NF-kappaB): potential implications on prostate cancer chemoprevention and therapy. J Pineal Res, 54:69-79. 22856547 10.1111/j.1600-079X.2012.01026.x

[60] Liu VWS, Yau WL, Tam CW, Yao KM, Shiu SYW (2017). Melatonin Inhibits Androgen Receptor Splice Variant-7 (AR-V7)-Induced Nuclear Factor-Kappa B (NF-kappaB) Activation and NF-kappaB Activator-Induced AR-V7 Expression in Prostate Cancer Cells: Potential Implications for the Use of Melatonin in Castration-Resistant Prostate Cancer (CRPC) Therapy. Int J Mol Sci, 18. 10.3390/ijms18061130 PMC5485954 28561752

[61] Macri E, Loda M (1998). Role of p27 in prostate carcinogenesis. Cancer Metastasis Rev, 17:337-344. 10453277 10.1023/a:1006133620914

[62] Tsihlias J, Kapusta LR, DeBoer G, Morava-Protzner I, Zbieranowski I, Bhattacharya N, et al. (1998). Loss of cyclin-dependent kinase inhibitor p27Kip1 is a novel prognostic factor in localized human prostate adenocarcinoma. Cancer Res, 58:542-548. 9458103

[63] Tam CW, Shiu SY (2011). Functional interplay between melatonin receptor-mediated antiproliferative signaling and androgen receptor signaling in human prostate epithelial cells: potential implications for therapeutic strategies against prostate cancer. J Pineal Res, 51:297-312. 21605164 10.1111/j.1600-079X.2011.00890.x

[64] Jung-Hynes B, Nihal M, Zhong W, Ahmad N (2009). Role of sirtuin histone deacetylase SIRT1 in prostate cancer. A target for prostate cancer management via its inhibition? J Biol Chem, 284:3823-3832. 19075016 10.1074/jbc.M807869200 PMC2635052

[65] Jung-Hynes B, Schmit TL, Reagan-Shaw SR, Siddiqui IA, Mukhtar H, Ahmad N (2011). Melatonin, a novel Sirt1 inhibitor, imparts antiproliferative effects against prostate cancer in vitro in culture and in vivo in TRAMP model. J Pineal Res, 50:140-149. 21062352 10.1111/j.1600-079X.2010.00823.x PMC3052633

[66] Rimler A, Culig Z, Levy-Rimler G, Lupowitz Z, Klocker H, Matzkin H, et al. (2001). Melatonin elicits nuclear exclusion of the human androgen receptor and attenuates its activity. Prostate, 49:145-154. 11582594 10.1002/pros.1129

[67] Rimler A, Culig Z, Lupowitz Z, Zisapel N (2002). Nuclear exclusion of the androgen receptor by melatonin. J Steroid Biochem Mol Biol, 81:77-84. 12127045 10.1016/s0960-0760(02)00050-x

[68] Sampson SR, Lupowitz Z, Braiman L, Zisapel N (2006). Role of protein kinase Calpha in melatonin signal transduction. Mol Cell Endocrinol, 252:82-87. 16697522 10.1016/j.mce.2006.03.033

[69] Mayo JC, Hevia D, Quiros-Gonzalez I, Rodriguez-Garcia A, Gonzalez-Menendez P, Cepas V, et al. (2017). IGFBP3 and MAPK/ERK signaling mediates melatonin-induced antitumor activity in prostate cancer. J Pineal Res, 62. 10.1111/jpi.12373 27736013

[70] Dieter P, Fitzke E, Duyster J (1993). BAPTA induces a decrease of intracellular free calcium and a translocation and inactivation of protein kinase C in macrophages. Biol Chem Hoppe Seyler, 374:171-174. 8387795 10.1515/bchm3.1993.374.1-6.171

[71] Lupowitz Z, Rimler A, Zisapel N (2001). Evaluation of signal transduction pathways mediating the nuclear exclusion of the androgen receptor by melatonin. Cell Mol Life Sci, 58:2129-2135. 11814062 10.1007/PL00000842 PMC11337335

[72] Newton AC (1995). Protein kinase C: structure, function, and regulation. J Biol Chem, 270:28495-28498. 7499357 10.1074/jbc.270.48.28495

[73] Xi SC, Tam PC, Brown GM, Pang SF, Shiu SY (2000). Potential involvement of mt1 receptor and attenuated sex steroid-induced calcium influx in the direct anti-proliferative action of melatonin on androgen-responsive LNCaP human prostate cancer cells. J Pineal Res, 29:172-183. 11034115 10.1034/j.1600-079x.2000.d01-64.x

[74] Saxena P, Trerotola M, Wang T, Li J, Sayeed A, Vanoudenhove J, et al. (2012). PSA regulates androgen receptor expression in prostate cancer cells. Prostate, 72:769-776. 21956655 10.1002/pros.21482 PMC3404455

[75] Kim J, Coetzee GA (2004). Prostate specific antigen gene regulation by androgen receptor. J Cell Biochem, 93:233-241. 15368351 10.1002/jcb.20228

[76] Sharifi N, Gulley JL, Dahut WL (2005). Androgen deprivation therapy for prostate cancer. JAMA, 294:238-244. 16014598 10.1001/jama.294.2.238

[77] Dai C, Heemers H, Sharifi N (2017). Androgen Signaling in Prostate Cancer. Cold Spring Harb Perspect Med, 7. 10.1101/cshperspect.a030452 PMC5580512 28389515

[78] Ross RW, Xie W, Regan MM, Pomerantz M, Nakabayashi M, Daskivich TJ, et al. (2008). Efficacy of androgen deprivation therapy (ADT) in patients with advanced prostate cancer: association between Gleason score, prostate-specific antigen level, and prior ADT exposure with duration of ADT effect. Cancer, 112:1247-1253. 18293426 10.1002/cncr.23304

[79] Sharifi N, Dahut WL, Steinberg SM, Figg WD, Tarassoff C, Arlen P, et al. (2005). A retrospective study of the time to clinical endpoints for advanced prostate cancer. BJU Int, 96:985-989. 16225513 10.1111/j.1464-410X.2005.05798.x

[80] Hotte SJ, Saad F (2010). Current management of castrate-resistant prostate cancer. Curr Oncol, 17 Suppl 2:S72-79. 20882137 10.3747/co.v17i0.718 PMC2935714

[81] Yuan X, Balk SP (2009). Mechanisms mediating androgen receptor reactivation after castration. Urol Oncol, 27:36-41. 19111796 10.1016/j.urolonc.2008.03.021 PMC3245883

[82] Litvinov IV, De Marzo AM, Isaacs JT (2003). Is the Achilles' heel for prostate cancer therapy a gain of function in androgen receptor signaling? J Clin Endocrinol Metab, 88:2972-2982. 12843129 10.1210/jc.2002-022038

[83] Lu C, Luo J (2013). Decoding the androgen receptor splice variants. Transl Androl Urol, 2:178-186. 25356377 10.3978/j.issn.2223-4683.2013.09.08 PMC4209743

[84] Hu R, Dunn TA, Wei S, Isharwal S, Veltri RW, Humphreys E, et al. (2009). Ligand-independent androgen receptor variants derived from splicing of cryptic exons signify hormone-refractory prostate cancer. Cancer Res, 69:16-22. 19117982 10.1158/0008-5472.CAN-08-2764 PMC2614301

[85] Sharp A, Coleman I, Yuan W, Sprenger C, Dolling D, Rodrigues DN, et al. (2019). Androgen receptor splice variant-7 expression emerges with castration resistance in prostate cancer. J Clin Invest, 129:192-208. 30334814 10.1172/JCI122819 PMC6307949

[86] Culig Z (2014). Proinflammatory cytokine interleukin-6 in prostate carcinogenesis. Am J Clin Exp Urol, 2:231-238. 25374925 PMC4219316

[87] Culig Z, Puhr M (2018). Interleukin-6 and prostate cancer: Current developments and unsolved questions. Mol Cell Endocrinol, 462:25-30. 28315704 10.1016/j.mce.2017.03.012

[88] Park JW, Hwang MS, Suh SI, Baek WK (2009). Melatonin down-regulates HIF-1 alpha expression through inhibition of protein translation in prostate cancer cells. J Pineal Res, 46:415-421. 19552765 10.1111/j.1600-079X.2009.00678.x

[89] Elzakra N, Kim Y (2021). HIF-1alpha Metabolic Pathways in Human Cancer. Adv Exp Med Biol, 1280:243-260. 33791987 10.1007/978-3-030-51652-9_17

[90] Carmeliet P (2005). VEGF as a key mediator of angiogenesis in cancer. Oncology, 69 Suppl 3:4-10. 16301830 10.1159/000088478

[91] Xiao L, Wang YC, Li WS, Du Y (2009). The role of mTOR and phospho-p70S6K in pathogenesis and progression of gastric carcinomas: an immunohistochemical study on tissue microarray. J Exp Clin Cancer Res, 28:152. 20003385 10.1186/1756-9966-28-152 PMC2797794

[92] Kwon HK, Bae GU, Yoon JW, Kim YK, Lee HY, Lee HW, et al. (2002). Constitutive activation of p70S6k in cancer cells. Arch Pharm Res, 25:685-690. 12433206 10.1007/BF02976945

[93] Laughner E, Taghavi P, Chiles K, Mahon PC, Semenza GL (2001). HER2 (neu) signaling increases the rate of hypoxia-inducible factor 1alpha (HIF-1alpha) synthesis: novel mechanism for HIF-1-mediated vascular endothelial growth factor expression. Mol Cell Biol, 21:3995-4004. 11359907 10.1128/MCB.21.12.3995-4004.2001 PMC87062

[94] Pyne NJ, Pyne S (2010). Sphingosine 1-phosphate and cancer. Nat Rev Cancer, 10:489-503. 20555359 10.1038/nrc2875

[95] Haase VH (2009). The VHL tumor suppressor: master regulator of HIF. Curr Pharm Des, 15:3895-3903. 19671042 10.2174/138161209789649394 PMC3622710

[96] Ader I, Brizuela L, Bouquerel P, Malavaud B, Cuvillier O (2008). Sphingosine kinase 1: a new modulator of hypoxia inducible factor 1alpha during hypoxia in human cancer cells. Cancer Res, 68:8635-8642. 18922940 10.1158/0008-5472.CAN-08-0917

[97] Ader I, Malavaud B, Cuvillier O (2009). When the sphingosine kinase 1/sphingosine 1-phosphate pathway meets hypoxia signaling: new targets for cancer therapy. Cancer Res, 69:3723-3726. 19383898 10.1158/0008-5472.CAN-09-0389

[98] Cho SY, Lee HJ, Jeong SJ, Lee HJ, Kim HS, Chen CY, et al. (2011). Sphingosine kinase 1 pathway is involved in melatonin-induced HIF-1alpha inactivation in hypoxic PC-3 prostate cancer cells. J Pineal Res, 51:87-93. 21392092 10.1111/j.1600-079X.2011.00865.x

[99] Macfarlane LA, Murphy PR (2010). MicroRNA: Biogenesis, Function and Role in Cancer. Curr Genomics, 11:537-561. 21532838 10.2174/138920210793175895 PMC3048316

[100] Sohn EJ, Won G, Lee J, Lee S, Kim SH (2015). Upregulation of miRNA3195 and miRNA374b Mediates the Anti-Angiogenic Properties of Melatonin in Hypoxic PC-3 Prostate Cancer Cells. J Cancer, 6:19-28. 25553085 10.7150/jca.9591 PMC4278911

[101] Scholzen T, Gerdes J (2000). The Ki-67 protein: from the known and the unknown. J Cell Physiol, 182:311-322. 10653597 10.1002/(SICI)1097-4652(200003)182:3<311::AID-JCP1>3.0.CO;2-9

[102] Ghosh PM, Malik SN, Bedolla RG, Wang Y, Mikhailova M, Prihoda TJ, et al. (2005). Signal transduction pathways in androgen-dependent and -independent prostate cancer cell proliferation. Endocr Relat Cancer, 12:119-134. 15788644 10.1677/erc.1.00835

[103] Paroni R, Terraneo L, Bonomini F, Finati E, Virgili E, Bianciardi P, et al. (2014). Antitumour activity of melatonin in a mouse model of human prostate cancer: relationship with hypoxia signalling. J Pineal Res, 57:43-52. 24786921 10.1111/jpi.12142

[104] Gandaglia G, Abdollah F, Schiffmann J, Trudeau V, Shariat SF, Kim SP, et al. (2014). Distribution of metastatic sites in patients with prostate cancer: A population-based analysis. Prostate, 74:210-216. 24132735 10.1002/pros.22742

[105] Chen PC, Tang CH, Lin LW, Tsai CH, Chu CY, Lin TH, et al. (2017). Thrombospondin-2 promotes prostate cancer bone metastasis by the up-regulation of matrix metalloproteinase-2 through down-regulating miR-376c expression. J Hematol Oncol, 10:33. 28122633 10.1186/s13045-017-0390-6 PMC5264454

[106] Gonzalez-Avila G, Sommer B, Mendoza-Posada DA, Ramos C, Garcia-Hernandez AA, Falfan-Valencia R (2019). Matrix metalloproteinases participation in the metastatic process and their diagnostic and therapeutic applications in cancer. Crit Rev Oncol Hematol, 137:57-83. 31014516 10.1016/j.critrevonc.2019.02.010

[107] Martin MD, Matrisian LM (2007). The other side of MMPs: protective roles in tumor progression. Cancer Metastasis Rev, 26:717-724. 17717634 10.1007/s10555-007-9089-4

[108] Kleiner DE, Stetler-Stevenson WG (1999). Matrix metalloproteinases and metastasis. Cancer Chemother Pharmacol, 43 Suppl:S42-51. 10357558 10.1007/s002800051097

[109] Gong Y, Chippada-Venkata UD, Oh WK (2014). Roles of matrix metalloproteinases and their natural inhibitors in prostate cancer progression. Cancers (Basel), 6:1298-1327. 24978435 10.3390/cancers6031298 PMC4190542

[110] Wu D, Huang P, Wang L, Zhou Y, Pan H, Qu P (2013). MicroRNA-143 inhibits cell migration and invasion by targeting matrix metalloproteinase 13 in prostate cancer. Mol Med Rep, 8:626-630. 23732700 10.3892/mmr.2013.1501

[111] Benbow U, Brinckerhoff CE (1997). The AP-1 site and MMP gene regulation: what is all the fuss about? Matrix Biol, 15:519-526. 9138284 10.1016/s0945-053x(97)90026-3

[112] Wang SW, Tai HC, Tang CH, Lin LW, Lin TH, Chang AC, et al. (2021). Melatonin impedes prostate cancer metastasis by suppressing MMP-13 expression. J Cell Physiol, 236:3979-3990. 33251599 10.1002/jcp.30150

[113] Tamarindo GH, Ribeiro DL, Gobbo MG, Guerra LHA, Rahal P, Taboga SR, et al. (2019). Melatonin and Docosahexaenoic Acid Decrease Proliferation of PNT1A Prostate Benign Cells via Modulation of Mitochondrial Bioenergetics and ROS Production. Oxid Med Cell Longev, 2019:5080798. 30728886 10.1155/2019/5080798 PMC6343140

[114] Rodriguez-Garcia A, Hevia D, Mayo JC, Gonzalez-Menendez P, Coppo L, Lu J, et al. (2017). Thioredoxin 1 modulates apoptosis induced by bioactive compounds in prostate cancer cells. Redox Biol, 12:634-647. 28391184 10.1016/j.redox.2017.03.025 PMC5385622

[115] Kabasakal L, Sener G, Balkan J, Dogru-Abbasoglu S, Keyer-Uysal M, Uysal M (2011). Melatonin and beta-glucan alone or in combination inhibit the growth of dunning prostatic adenocarcinoma. Oncol Res, 19:259-263. 21776821 10.3727/096504011x13021877989748

[116] Neri B, de Leonardis V, Gemelli MT, di Loro F, Mottola A, Ponchietti R, et al. (1998). Melatonin as biological response modifier in cancer patients. Anticancer Res, 18:1329-1332. 9615811

[117] Ge D, Dauchy RT, Liu S, Zhang Q, Mao L, Dauchy EM, et al. (2013). Insulin and IGF1 enhance IL-17-induced chemokine expression through a GSK3B-dependent mechanism: a new target for melatonin's anti-inflammatory action. J Pineal Res, 55:377-387. 24033914 10.1111/jpi.12084 PMC3797167

[118] Abbas T, Dutta A (2009). p21 in cancer: intricate networks and multiple activities. Nat Rev Cancer, 9:400-414. 19440234 10.1038/nrc2657 PMC2722839

[119] Suh J, Rabson AB (2004). NF-kappaB activation in human prostate cancer: important mediator or epiphenomenon? J Cell Biochem, 91:100-117. 14689584 10.1002/jcb.10729

[120] Yue J, Lopez JM (2020). Understanding MAPK Signaling Pathways in Apoptosis. Int J Mol Sci, 21. 10.3390/ijms21072346 PMC7177758 32231094

[121] Balmanno K, Cook SJ (2009). Tumour cell survival signalling by the ERK1/2 pathway. Cell Death Differ, 16:368-377. 18846109 10.1038/cdd.2008.148

[122] Wagner EF, Nebreda AR (2009). Signal integration by JNK and p38 MAPK pathways in cancer development. Nat Rev Cancer, 9:537-549. 19629069 10.1038/nrc2694

[123] Joo SS, Yoo YM (2009). Melatonin induces apoptotic death in LNCaP cells via p38 and JNK pathways: therapeutic implications for prostate cancer. J Pineal Res, 47:8-14. 19522739 10.1111/j.1600-079X.2009.00682.x

[124] Cavalieri E, Chakravarti D, Guttenplan J, Hart E, Ingle J, Jankowiak R, et al. (2006). Catechol estrogen quinones as initiators of breast and other human cancers: implications for biomarkers of susceptibility and cancer prevention. Biochim Biophys Acta, 1766:63-78. 16675129 10.1016/j.bbcan.2006.03.001

[125] Markushin Y, Gaikwad N, Zhang H, Kapke P, Rogan EG, Cavalieri EL, et al. (2006). Potential biomarker for early risk assessment of prostate cancer. Prostate, 66:1565-1571. 16894534 10.1002/pros.20484

[126] Zahid M, Gaikwad NW, Rogan EG, Cavalieri EL (2007). Inhibition of depurinating estrogen-DNA adduct formation by natural compounds. Chem Res Toxicol, 20:1947-1953. 18039013 10.1021/tx700269s

[127] Kazazian HH Jr, Moran JV (2017). Mobile DNA in Health and Disease. N Engl J Med, 377:361-370. 28745987 10.1056/NEJMra1510092 PMC5980640

[128] Brennecke J, Malone CD, Aravin AA, Sachidanandam R, Stark A, Hannon GJ (2008). An epigenetic role for maternally inherited piRNAs in transposon silencing. Science, 322:1387-1392. 19039138 10.1126/science.1165171 PMC2805124

[129] Castro-Diaz N, Ecco G, Coluccio A, Kapopoulou A, Yazdanpanah B, Friedli M, et al. (2014). Evolutionally dynamic L1 regulation in embryonic stem cells. Genes Dev, 28:1397-1409. 24939876 10.1101/gad.241661.114 PMC4083085

[130] Schulz WA (2006). L1 retrotransposons in human cancers. J Biomed Biotechnol, 2006:83672. 16877821 10.1155/JBB/2006/83672 PMC1559935

[131] Miki Y, Nishisho I, Horii A, Miyoshi Y, Utsunomiya J, Kinzler KW, et al. (1992). Disruption of the APC gene by a retrotransposal insertion of L1 sequence in a colon cancer. Cancer Res, 52:643-645. 1310068

[132] Rodic N, Sharma R, Sharma R, Zampella J, Dai L, Taylor MS, et al. (2014). Long interspersed element-1 protein expression is a hallmark of many human cancers. Am J Pathol, 184:1280-1286. 24607009 10.1016/j.ajpath.2014.01.007 PMC4005969

[133] deHaro D, Kines KJ, Sokolowski M, Dauchy RT, Streva VA, Hill SM, et al. (2014). Regulation of L1 expression and retrotransposition by melatonin and its receptor: implications for cancer risk associated with light exposure at night. Nucleic Acids Res, 42:7694-7707. 24914052 10.1093/nar/gku503 PMC4081101

[134] Hu CD, Choo R, Huang J (2015). Neuroendocrine differentiation in prostate cancer: a mechanism of radioresistance and treatment failure. Front Oncol, 5:90. 25927031 10.3389/fonc.2015.00090 PMC4396194

[135] Huang J, Wu C, di Sant'Agnese PA, Yao JL, Cheng L, Na Y (2007). Function and molecular mechanisms of neuroendocrine cells in prostate cancer. Anal Quant Cytol Histol, 29:128-138. 17672372

[136] Amorino GP, Parsons SJ (2004). Neuroendocrine cells in prostate cancer. Crit Rev Eukaryot Gene Expr, 14:287-300. 15663358 10.1615/critreveukaryotgeneexpr.v14.i4.40

[137] Butler W, Huang J (2021). Neuroendocrine cells of the prostate: Histology, biological functions, and molecular mechanisms. Precis Clin Med, 4:25-34. 33842835 10.1093/pcmedi/pbab003 PMC8023015

[138] Aggarwal R, Huang J, Alumkal JJ, Zhang L, Feng FY, Thomas GV, et al. (2018). Clinical and Genomic Characterization of Treatment-Emergent Small-Cell Neuroendocrine Prostate Cancer: A Multi-institutional Prospective Study. J Clin Oncol, 36:2492-2503. 29985747 10.1200/JCO.2017.77.6880 PMC6366813

[139] Clermont P-L, Ci X, Pandha H, Wang Y, Crea F (2019). Treatment-emergent neuroendocrine prostate cancer: molecularly driven clinical guidelines. International Journal of Endocrine Oncology, 6:IJE20.

[140] Parimi V, Goyal R, Poropatich K, Yang XJ (2014). Neuroendocrine differentiation of prostate cancer: a review. Am J Clin Exp Urol, 2:273-285. 25606573 PMC4297323

[141] Hirano D, Okada Y, Minei S, Takimoto Y, Nemoto N (2004). Neuroendocrine differentiation in hormone refractory prostate cancer following androgen deprivation therapy. Eur Urol, 45:586-592; discussion 592. 15082200 10.1016/j.eururo.2003.11.032

[142] Sainz RM, Mayo JC, Tan DX, Leon J, Manchester L, Reiter RJ (2005). Melatonin reduces prostate cancer cell growth leading to neuroendocrine differentiation via a receptor and PKA independent mechanism. Prostate, 63:29-43. 15378522 10.1002/pros.20155

[143] Quiros I, Sainz RM, Hevia D, Garcia-Suarez O, Astudillo A, Rivas M, et al. (2009). Upregulation of manganese superoxide dismutase (SOD2) is a common pathway for neuroendocrine differentiation in prostate cancer cells. Int J Cancer, 125:1497-1504. 19507253 10.1002/ijc.24501

[144] Terry S, Beltran H (2014). The many faces of neuroendocrine differentiation in prostate cancer progression. Front Oncol, 4:60. 24724054 10.3389/fonc.2014.00060 PMC3971158

[145] Rodriguez-Garcia A, Mayo JC, Hevia D, Quiros-Gonzalez I, Navarro M, Sainz RM (2013). Phenotypic changes caused by melatonin increased sensitivity of prostate cancer cells to cytokine-induced apoptosis. J Pineal Res, 54:33-45. 22738066 10.1111/j.1600-079X.2012.01017.x

[146] Reppert SM, Weaver DR (2002). Coordination of circadian timing in mammals. Nature, 418:935-941. 12198538 10.1038/nature00965

[147] Jung-Hynes B, Huang W, Reiter RJ, Ahmad N (2010). Melatonin resynchronizes dysregulated circadian rhythm circuitry in human prostate cancer cells. J Pineal Res, 49:60-68. 20524973 10.1111/j.1600-079X.2010.00767.x PMC3158680

[148] Sedighi Pashaki A, Mohammadian K, Afshar S, Gholami MH, Moradi A, Javadinia SA, et al. (2021). A Randomized, Controlled, Parallel-Group, Trial on the Effects of Melatonin on Fatigue Associated with Breast Cancer and Its Adjuvant Treatments. Integr Cancer Ther, 20:1534735420988343. 33543655 10.1177/1534735420988343 PMC7868453

[149] Palmer ACS, Zortea M, Souza A, Santos V, Biazus JV, Torres ILS, et al. (2020). Clinical impact of melatonin on breast cancer patients undergoing chemotherapy; effects on cognition, sleep and depressive symptoms: A randomized, double-blind, placebo-controlled trial. PLoS One, 15:e0231379. 32302347 10.1371/journal.pone.0231379 PMC7164654

[150] Meng X, Li Y, Li S, Zhou Y, Gan RY, Xu DP, et al. (2017). Dietary Sources and Bioactivities of Melatonin. Nutrients, 9. 10.3390/nu9040367 PMC5409706 28387721

[151] Lissoni P, Barni S, Mandala M, Ardizzoia A, Paolorossi F, Vaghi M, et al. (1999). Decreased toxicity and increased efficacy of cancer chemotherapy using the pineal hormone melatonin in metastatic solid tumour patients with poor clinical status. Eur J Cancer, 35:1688-1692. 10674014 10.1016/s0959-8049(99)00159-8

[152] Wang L, Wang C, Choi WS (2022). Use of Melatonin in Cancer Treatment: Where Are We? Int J Mol Sci, 23. 10.3390/ijms23073779 PMC8998229 35409137

[153] Cerea G, Vaghi M, Ardizzoia A, Villa S, Bucovec R, Mengo S, et al. (2003). Biomodulation of cancer chemotherapy for metastatic colorectal cancer: a randomized study of weekly low-dose irinotecan alone versus irinotecan plus the oncostatic pineal hormone melatonin in metastatic colorectal cancer patients progressing on 5-fluorouracil-containing combinations. Anticancer Res, 23:1951-1954. 12820485

[154] Lissoni P, Barni S, Meregalli S, Fossati V, Cazzaniga M, Esposti D, et al. (1995). Modulation of cancer endocrine therapy by melatonin: a phase II study of tamoxifen plus melatonin in metastatic breast cancer patients progressing under tamoxifen alone. Br J Cancer, 71:854-856. 7710954 10.1038/bjc.1995.164 PMC2033724

[155] Gonzalez A, Alonso-Gonzalez C, Gonzalez-Gonzalez A, Menendez-Menendez J, Cos S, Martinez-Campa C (2021). Melatonin as an Adjuvant to Antiangiogenic Cancer Treatments. Cancers(Basel), 13. 10.3390/cancers13133263 PMC8268559 34209857

[156] Seely D, Legacy M, Auer RC, Fazekas A, Delic E, Anstee C, et al. (2021). Adjuvant melatonin for the prevention of recurrence and mortality following lung cancer resection (AMPLCaRe): A randomized placebo controlled clinical trial. EClinicalMedicine, 33:100763. 33681747 10.1016/j.eclinm.2021.100763 PMC7930365

[157] Foley HM, Steel AE (2019). Adverse events associated with oral administration of melatonin: A critical systematic review of clinical evidence. Complement Ther Med, 42:65-81. 30670284 10.1016/j.ctim.2018.11.003

